# The Effect of Spiritual Counseling on Hope in Patients with Multiple Sclerosis: A Randomized Clinical Trial

**DOI:** 10.30476/ijcbnm.2021.88605.1523

**Published:** 2021-10

**Authors:** Mohammad Afshar, Zohreh Sadat, Mohhammad Bagheri

**Affiliations:** 1 Trauma Nursing Research Center, Kashan University of Medical Sciences, Kashan, Iran; 2 Department of Psychiatric Nursing, Kashan University of Medical Sciences, Kashan, Iran

**Keywords:** Counseling, Hope, Multiple sclerosis, Spiritual therapies

## Abstract

**Background:**

Spiritual practices have recently emerged as beneficial to both mental and physical health. The present study was conducted to determine the effect of spiritual counseling on hope among patients with Multiple Sclerosis (MS).

**Methods:**

This single blind randomized controlled clinical trial was conducted on the patients with MS in April-June 2020, Kashan, Iran. 50 patients were randomly assigned to two 25-member groups. The patients in the intervention group participated in eight 60-minute spiritual counseling program. They were asked to fill out the demographic information questionnaire before the intervention and Herth Hope Index (HHI) before, immediately (in the 4th week of the study) and a month after the intervention (in the 8th week of the study). Data were analyzed using Chi-square, independent samples t-test and repeated measures ANOVA in SPSS version 16. The significance level was considered P<0.05.

**Results:**

The results showed that the differences between the two groups before the intervention were not statistically significant in terms of demographic variables (P>0.05) and the mean score of hope (P=0.61). However, the total mean score of HHI in the intervention group was significantly different from the control group immediately and a month after the intervention (44.95±1.42 VS. 31.66±2.45 and 43.25±1.84 VS. 30.58±2.24), respectively (P<0.001). According to the results of the repeated measures ANOVA, the level of hope and its dimensions significantly changed in the intervention group over time (P<0.001).

**Conclusion:**

The results of the present study showed that spiritual counseling promoted the hope score in patients with MS. It is recommended that spiritual counseling should be use as a complementary therapy along with other counseling treatments to increase hope in patients with MS.

**Trial Registration Number:**

IRCT20190819044567N

## INTRODUCTION

Multiple sclerosis (MS) is a chronic, progressive disease of the central nervous system. The myelin sheath, which insulates the nerve cell axons and enhances transmission of impulses from the brain to other parts of the body is damaged. As a result of this demyelination process, messages that pass along the nerve are transmitted with delay or become blocked. ^1^ There are about 2.5 million people suffering from MS around the world. ^2^ Iran is considered to be located in a medium prevalence zone for MS and its rates have dramatically increased in the past 20 years. ^3^ Information has not been published on the prevalence and incidence of the disease in Kashan city (located in province of Isfahan, Iran). However, the highest prevalence of MS (89/100,000 people) has been reported in Isfahan city compared to other cities of Iran. ^4^

There is not any certain treatment for MS, and its treatment can be achieved only by comprehensive attention. ^5^ This attention requires holistic view on human nature, which has different aspects of physical, mental, social, and spiritual. ^6^ Spirituality is a multidimensional and compound concept which has cognitive, emotional and behavioral features, and it typically involves a search for meaning in life. ^7^ Spiritual health is known as the most important issue in helping to increase the patients’ skills to cope with illnesses. ^8^ Spiritual health is so important that the World Health Organization announced the spiritual aspect as one of the dimensions of health along with physical, mental and social well-being. ^9^

Spirituality is the beliefs related to personal subjective sense of existential communications including beliefs that identify a person’s place in the world, reflect relations with others, acknowledge a higher power, and lead to spiritual practices. ^10^ Researchers demonstrated that most of the patients believe that the spirituality plays an important part in their lives and medical care, and also they believe that the physician and nurses should pay attention to this factor in their health care. ^11^ Furthermore, perception of the effect of spirituality on the care of patients can affect their survival. ^12^ In Iranian-Islamic culture, spiritual and religious beliefs play an important role in life quality, especially in critical conditions. Providing spiritual care to the patients and their family can improve the recovery rate, emotional well-being, hope, patient-nurse relationship, and create goal in life. ^13^ Spiritual care is an essential factor of comprehensive nursing which has already been combined into nursing education and practice. In addition, the ability to provide spiritual care to patients is progressively considered a major job-related skill for nurses. ^14 , 15^

Patients with MS have high levels of psychological distress in comparison with healthy people. Nearly 60% of these patients experience at least one period of mental health problems during the illness. The most important mental disorders in MS include cognitive disorders, emotional changes, depression, and anxiety, which significantly affect the patients’ well-being. ^16^ Difficulties of MS can also reduce hope that is the main component of life satisfaction; also hope is defined as the belief in better quality of life in the future. ^17 , 18^ Hope is thought as one of the cores of human adjustment to the difficulties and serious diseases. ^19^ The low levels of hope in patients with MS leads to a loss of motivation to achieve their goals. ^18^ However, identifying the protective factors such as hope in patients with MS can play an significant role in addressing the related challenges and goals and reduce the effect of adverse outcomes of this illness. ^5^

Spiritual group counseling significantly has increased the hope of disabled people such as patients with breast cancer, diabetes, and chronic renal failure. ^20 - 22^ However, to the best of our knowledge, a similar study has not been conducted in other countries, but two studies examined the effect of spirituality counseling on hope in patients with MS in Iran (in Tehran and Isfahan cities, respectively). The results of two studies showed that spiritual health education could increase the score of hope. ^23 , 24^ In both studies, follow-up test was not performed to determine the stability of the results for a long time. In addition, in the second study, all the participants were women and the sample size in both studies was 15 people in each group. Therefore, we decided to assess the stability of the effect of the intervention by performing a follow-up test one month after the intervention; the participants consisted of men and women, and more sample size was considered. 

Given the lack of sufficient studies about the role of spirituality on hope in patients with MS and its important role in nursing services and also different approaches of spiritual counseling in Iranian-Islamic culture, more investigation is required. Therefore, the present study aimed to investigate the effect of spiritual counseling on hope in patients with MS.

## MATERIALS AND METHODS

This study is a single blind randomized controlled clinical trial conducted on 50 out of 128 MS patients who were registered in the Kashan MS Society (KMSS) from April-June 2020.

The sample size was calculated using the formula below. with α=0.05, β=0.10 and the mean (mean 1=36.48, mean 2=31.58) and variance($\sigma_{1}^{2}=6.20$ and $\sigma_{2}^{2}=3.26$ ) based on the results of a previous study. ^25^ At least, the sample size of 22 patients was calculated for each study group. Generally, 25 participants were selected for each group considering the possible attrition of 10%.

$n=\frac{{\left(z_{1-\frac{\alpha }{2}}+z_{1-\beta })(\sigma_{1}^{2}+\sigma_{2}^{2}\right)}^{2}}{{\left(\mu_{1}-\mu_{2}\right)}^{2}}$

$n=\frac{{\left(1.96+1.28\right)}^{2}(6.20^{2}+3.26^{2})}{{\left(\mathrm{36 .48}-\mathrm{31 .58}\right)}^{2}}=22$

Inclusion criteria were patients with mild or moderate disability (walking without any aids), MS diagnosed at least in the last 3 months by the neurologist, no diagnosis of underlying diseases and cognitive disorders, ability to read and write, Iranian nationality, and residence in Kashan city. 

The subjects were excluded if they missed more than two counseling sessions, refused to continue participation, were hospitalized or got out of access, and experienced unfortunate events such as loved one’s unexpected death during the study.

The study instruments included the demographic questionnaire (age, sex, marital status, educational level, duration of illness, job) and HHI. The HHI is a 12-item questionnaire scored from 1 to 4, where 1 is ‘strongly disagree’ and 4 is ‘strongly agree. The total score of this questionnaire varies from 12 to 48; the higher the score, the higher the level of hope. The tool develops and evaluates psychometric characteristics to assess hope in adults in clinical settings by Herth, 1992. ^26^ The developer of the tool has described excellent psychometric features: Chronbach’s alpha coefficient of 0.97, test-retest reliability (2 weeks) of 0.91, concurrent validity with the Hope Herth Scale of 0.92, and the Existential Well Being Scale of 0.84. Divergent validity was supported using the Hopelessness Scale (r=-073). Construct validity through exploratory factor analysis revealed that the index had three factors included: temporality and future formed from four items (1, 2, 6, 11), positive readiness and expectancy formed from four items (4, 7, 10, 12), and interconnectedness formed from four items (3, 5, 8, 9). ^26^ Abdi et al. conducted the psychometric analysis of Iranian version of HHI. The reliability of the HHI Persian version was assessed using internal consistency (Chronbach’s alpha coefficient 0.67), and concurrent validity was assessed by calculating the correlations of the HHI and the Miller Hope Scale (r=0.62). ^27^ Also in a study by Baljani et al., content validity of the questionnaire was confirmed by nursing instructors and its reliability was confirmed using internal consistency (Chronbach’s alpha coefficient of 0.82). ^28^ In the present study, the Cronbach’s alpha was estimated 0.94, which indicates the good reliability of the HHI.

**Figure 1:**
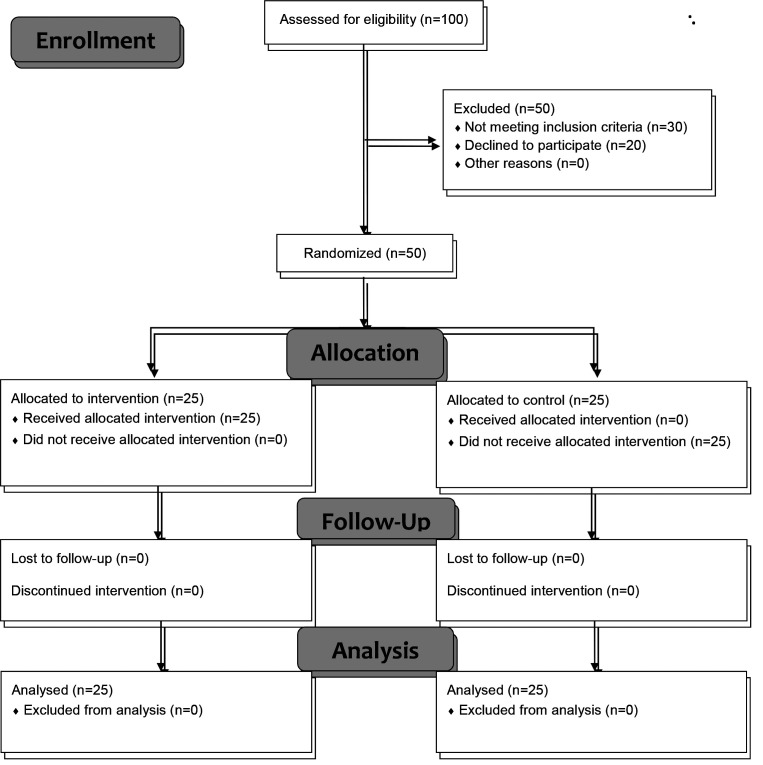
CONSORT Flow chart of the participants

A list was preapared of the names and phone numbers of the total MS patients recruited in KMSS (n=128). Then, all the patients were contacted by phone, 100 of whom responded to calls and were evaluated for eligibility. Among them, 30 patients did not meet the inclusion criteria and 20 declined to participate in the study (Figure 1). Subsequently 50 MS patients were allocated by the study supervisor into two equal (n=25) study groups using an online block randomization software ^29^ in five blocks of ten with unique randomization code. Then, the code and name of the groups for each patient were written on small cards and put in small opaque envelopes by the first researcher. Envelopes were arranged in order and provided to the research assistant (Psychiatric nurse who held the counseling classes). The assistant picked and unpacked an envelope and recommended the predetermined group. Thus, the subjects were assigned to one of the intervention or control groups. All the patients were invited to sign written informed consents at the place of holding classes. They were then introduced to the first researcher to complete two parts of a questionnaire which consisted of demographic and HHI at the beginning of the study. The patients in the intervention group participated in eight 60-minute sessions of spiritual counseling program. Sessions were held in the amphitheater of Kashan Naghavi hospital for two times per week for one month. The patients in the control group received no intervention. However, routine care was provided for both groups and included referring patients for medication and paramedical tests. Sometimes, they were helped to get free medication through charitable contributions and provided with information about MS. It should be noted that no training classes were held during the research period. Participants of the intervention group were informed about all the necessary information such as the total number of the sessions, time frequency and location, the content of each session, and the expected assignments. The content of the counseling program was derived from the instruction presented in the study of Shariatnia et al. and the contents of each session were approved by the faculty members and related experts ^30^ (Table 1). The counseling program was presented in the form of lectures, questions and answers and group discussion by a qualified psychiatric nurse expert in techniques of counseling and communication (research assistance). At the end of each session, the patients were asked to do the related homework and present them in the next session. The patients were invited by phone to complete the HHI questionnaire immediately (in 4th week of the study) and a month after the end of the counseling program (in the 8th week of the study) at the place of holding classes. As the study intervention was a consulting program, blinding of patients and instructors (research assistance) was impossible. However, the first researcher who collected the data and the statistician who analyzed the data were blind to the study groups. It should be noted that the participants in the intervention group were asked not to explain the content of the program to other participants. All the contents were provided for the control group at the end of the study.

**Table 1:** Content of spiritual counseling sessions

Sessions	Aim	Content
First session	Introduction, self-awareness, and recognition of strengths and weaknesses, needs, desires of human	Familiarizing the subjects with the purpose of research
		Discussion about self-awareness skill as a contributing factor to success and happiness in life
		Providing meaning to life and determining the purpose and impact of goals in life
		Explaining the need to know the personal strengths.
		Having a true understanding of personal disabilities and limitations.
		Accepting the responsibility of their own mistakes and learning from them.
Second session	Acceptance of monotheistic principles, prophecy, human right to determine their own destiny. the effect of the spiritual dimension in human life	Discussion about the stability of God’s willpower based on the Causal Argument.
		Discussion about the philosophy of the illness and life difficulties.
		Explaining the key role of hope and quality of life in physical and psychological dimensions of health.
		Defining the quality of life and hope for the future.
		Discussion about spirituality contributes to positive attitudes and helps people deal with unpleasant events in life
		Discussion about the role of spirituality principles in solving problems and preventing the problems such as hopeless
		Discussion about spiritual coping strategies
Third session	Repentance and confession of sins to God, the role of trust and faith in God in life, acceptance of own weaknesses and gaining knowledge about the consequences of not accepting them	Knowing about the God’s unconditional love for all the creatures and God’s awareness of the human mistakes
		Discussions about the role of trust and faith in God in life satisfaction
		Knowing about the God’s promise in the humans’ forgiveness.
		Knowing about the need of reforming the past to move towards the future.
		Making an effort to reach the peace and hope to improve the life
Fourth session	Releasing negative emotions caused by illness or past mistakes, control the stress and coping will	Know your feelings and the ways to express them.
		Express the repressed and undesirable emotions.
		Spend your mental energy in constructive activities and fight against the disease.
		Control the stress to adjust gradually and deal with negative emotions.
		Have a goal, as a basic factor for enjoying life despite illness.
		Expressing the philosophy of difficulties in life and presenting problem solving with a spiritual approach
Fifth session	Strengthening self-confidence and spiritual growth. Trying to remove the past negative thoughts and behavioral characteristics	Gain a deep knowledge of yourself, in order to realize your great hidden abilities.
		Recognize your weaknesses and try to eliminate them.
		Communication with people and use people skills through knowing them and their abilities.
		Gain benefit of the hope and not giving up in disappointing situations.
		Believe in your abilities and avoid feelings of inferiority.
		Avoid comparing yourself with others.
		Thinking about the philosophy of illness as a contributor to spiritual growth.
Sixth session	Development of spiritual beliefs and positive thinking and behaviors such as forgiveness to have a better life	Teaching how to control the thoughts according to their extraordinary constructive or destructive power.
		Teaching how to reach spiritual growth through positive thinking and rational actions.
		Having an optimistic outlook toward undesirable events using, according to a bit of Saadi, “Where one door shuts another opens
		Following the behavioral habits of successful people with high level of spirituality.
		Discussing about spiritual behaviors that are most useful for own and others
		Discussions about forgiveness and its effects and benefits
Seventh session	Empowering the individuals to solve their own and other people’s problems, thanksgiving and prayer	Discussion about psycho-cognitive empowerment, which includes five dimensions: Competence, Trust, Effectiveness, Autonomy, Meaningfulness
		Discussions about thanksgiving and its effects and benefits in life
		Discussions about praying and its effects and benefits in life.
Eighth session	Review and summarize the course contents	Learning about the role of the spiritual dimensions on development of human life
		Believing that the trust and faith in God lead to peace and satisfaction in life
		Learning about problem solving with a spiritual approach
		Believing that the purpose of human life is to know all the potential abilities and develop them
		Learning about the role of thanksgiving and prayer in life satisfaction
		Learning to take the responsibility in order to react to the tragic events of life appropriately using spiritual approach
		Learning continuous self-evaluation and monitoring the inner sensual desires.
		Learning how to control psychological reactions as an effective factor in physical illnesses using spiritual coping strategies
		Learning about the benefits of the forgiveness and helping the people in life

Data were analyzed using SPSS software version 16 (SPSS Inc., Chicago, IL, USA). The normality of the quantitative variables was tested using indices of skewness and kurtosis in the range between -2 and +2. Descriptive statistics (frequency, percentage, mean and standard deviation) were used to describe and classify the data. Chi-square test was used to compare qualitative variables, and independent samples t-test was used to compare quantitative demographic variables in the two study groups. To compare the means of hope score between the groups, in each time period and over the time, we used independent samples t-test and repeated measures ANOVA, respectively. The significance level was considered P<0.05.

The present study was registered in Iranian Registry of Clinical Trials center,a special code was received, and the Ethics Committee of Kashan University of Medical Sciences approved the study (Code of ethics=IR.KAUMS.NUHEPM.REC.1398.062). The participants were ensured about the confidentiality and all the questionnaires were kept anonymous. All participants were informed about voluntary participation. They did not have to pay for attending the counseling program and were allowe to withdraw from the study whenever they wished.

## RESULTS

According to the results, the range of participant’s age was 23-48 years and nearly half of them were over 35 years old. The mean age of the intervention group was 33.40±6.92 years and that of the control group was 36.08±6.21 years. The mean duration of illness (in months) in the intervention and control groups was 40.88±22.20 and 44.88±18.86, respectively. The results of independent t-test did not indicate a significant difference between the two groups in terms of age and duration of illness (P=0.15 and P=0.49), respectively. The results of Chi-square test did not indicate a significant difference between the two groups in terms of demographic variables including sex, marital status, level of education, and job (P>0.05) (Table 2).

**Table 2:** Demographic characteristics of the patients in the intervention and control groups

Variable	Intervention	Control	P value*
	(N=25)	(N=25)	
	N(%)	N(%)	
Sex			
Female	17(68)	15(60)	0.55
Male	8(32)	10(40)	
Educational level			
High school or less	2(8)	6(24)	0.28
Diploma	6(24)	6(24)	
Higher education	17(68)	13(52)	
Marital status			
Married	6(24)	9(36)	0.80
Single	13(52)	10(40)	
Other (divorced, widow)	6(24)	6(24)	
Job			
Retired	2(8)	2(8)	0.98
Non-governmental	9(36)	8(32)	
Worker	7(28)	7(28)	
House wife	7(28)	8(32)	

* Chi-square

**Figure 2:**
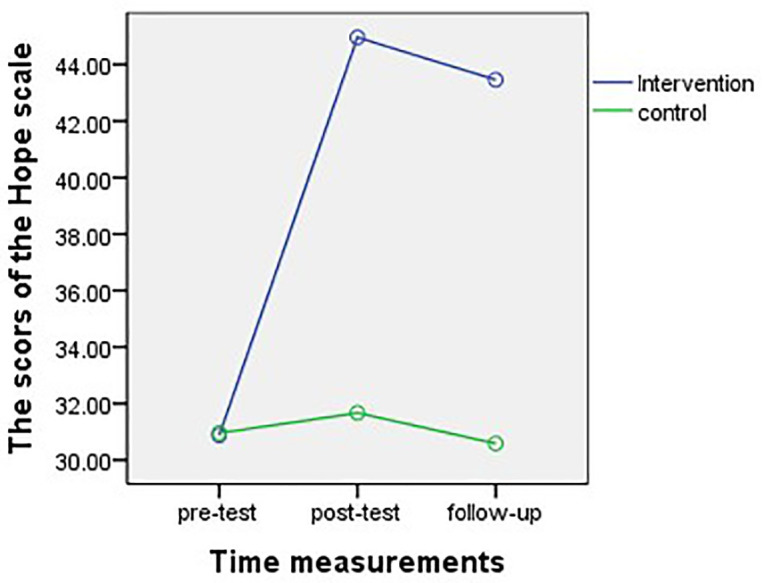
The mean scores of hope in Multiple Sclerosis patients in the intervention and control groups across the three measurement times

The mean scores of hope and its dimensions are presented in Table 3. The results of independent sample t-test did not show a significant difference between the groups before the intervention. Results showed that the mean score of hope and its dimensions in the intervention group was significantly different from the control group immediately and a month after the intervention (P<0.001). According to the results of the repeated measures ANOVA, the level of hope and its dimensions significantly changed in the intervention group over time (P<0.001), and the analysis of interaction effects showed a significant interaction between the group and time (P<0.001) (Table 3). As shown in Figure 2, the mean scores of hope in the intervention group increased over time.

**Table 3:** Comparisons of the mean hope scores and its dimensions at three measurement time points (start of study, fourth and eighth weeks after start of the intervention)

Variable	Intervention (N=25)	Control (N=25)	P value*	P value**		
	Mean±SD	Mean±SD		Group effect	Time effect	Group*Time effect
Total hope (12-48 score)						
Start of the study (T1)	30.88±1.61	31.12±1.71	P=0.61	P<0.001	P<0.001	P<0.001
Fourth week (T2)	44.95±1.42	31.66±2.45	P<0.001						
Eighth week (T3)	43.25±1.84	30.58±2.24	P<0.001						
P value**	P<0.001	0.08							
Dimension of temporality and future (4-16 score)						
Start of the study (T1)	10.35±1.41	10.16±1.21	0.59	P<0.001	P<0.001	P<0.001
Fourth week (T2)	14.83±0.84	10.50±1.21	P<0.001			
Eighth week (T3)	14.29±0.99	10.33±1.20	P<0.001			
P value**	P<0.001	0.21				
Dimension of positive readiness and expectancy (4-16 score)						
Start of the study (T1)	10.36±1.07	10.76±1.20	0.22	P<0.001	P<0.001	P<0.001
Fourth week (T2)	15.20±0.83	10.75±1.45	P<0.001			
Eighth week (T3)	14.35±1.20	10.12±1.26	P<0.001			
P value**	P<0.001	0.12				
Dimension of interconnectedness (4-16 score)						
Start of the study (T1)	10.16±1.34	10.20±1.29	0.91	P<0.001	P<0.001	P<0.001
Fourth week (T2)	14.91±0.92	10.40±1.38	P<0.001			
Eighth week (T3)	14.91±0.97	10.12±1.19	P<0.001			
P value**	P<0.001	0.27				

* Independent t-test;

** Repeated Measures ANOVA

## DISCUSSION

The present study demonstrates that the spiritual counseling increases hope in patients with MS. As a reason for this finding, it could be acknowledged that this kind of counseling helps the patients to feel the presence of God and benefit from God’s help and guidance in their lives. This makes them hopeful and they are assured that God will support them whenever they need. ^31^

In line with the results of the present study, a clinical trial study was performed on MS patients in Tehran. Spiritual health group teaching was held for the experimental group. Results showed that spiritual teaching made a positive effect on the score of hope in the experimental group. ^24^ An experimental study was performed on the women with MS who referred to MS Society of Iran in Tehran. Experimental groups received spiritual-religious care. Results showed the mean depression score was decreased significantly in the experimental group after the intervention. ^32^ In these studies, duration of the intervention, stage of MS, and sample size were similar to the present study. 

In line with the present results, a clinical trial was performed on hemodialysis patients. The intervention group received spirituality counseling. Results showed a significant difference between hope scores before and after counseling in the intervention group. ^25^ In this study, the method of intervention and the scale of hope assessment were similar to that of the present study. However, in their study the scores of the dimensions of hope were not reported. Another experimental study was performed to determine the effect of spiritual therapy on hope in women with breast cancer. Patients who referred to Firoozgar Cancer Center in Tehran were selected. The intervention group underwent spiritual therapy. The results showed that spiritual therapy had a positive effect on the score of hope in cancer patients. ^33^ A study examined the effect of group spirituality therapy on hope in breast cancer females at Omid hospital in Isfahan city. The results showed that the mean scores of hope in life in the experimental group increased significantly after the intervention. ^34^ A study was carried out on colorectal cancer patients in Kerman, Iran. Spiritual therapy was carried out on the experimental group. The study results showed that spiritual group therapy improved the scores of hope significantly. ^35^ In three current studies, similar to the present one, the patients had diseases that probably had subsequent consequences, such as depression and hopelessness. In addition, the number of spiritual counseling sessions and the sample size were similar to those of the present study. 

Contrary to the results of the present study, several studies reported no significant relationship between the hope and spirituality. ^36 - 38^ In this regard, a descriptive correlational study was performed to determine the score of hope and related factors among Italian hospitalized cancer patients. Results showed hope was positively correlated with several factors such as quality of life, self-esteem, coping, adjustment to the illness and well-being and negatively correlated with anxiety, depression and boredom during hospitalization. No statistically significant relationships were found between the overall hope score and socio-demographic characteristics, illness stage, religiosity, and spirituality. In this study, the spiritual intervention was not preformed to determine the effect of spirituality on hope; also, in their study the patients were hospitalized due to cancer. ^36^ A systematic review and meta-analysis study was performed to determine the effect of spiritual interventions on the quality of life among cancer patients. Several trials were included in the meta‐analysis. Overall, the study showed a moderate effect of spiritual interventions after the intervention on the quality of life. Meta‐analysis three to six months after the intervention showed a small insignificant effect of spiritual interventions on the quality of life. Authors conclude that further research is needed to understand how spiritual interventions could contribute to a long‐term effect of increasing or maintaining quality of life. ^37^ It should be noted that in the present study, the long-term effects of spiritual counseling were not investigated. A randomized controlled trial study was performed to determine spiritual reminiscence therapy in reducing loneliness, anxiety and depression for older people living in a residential aged care facility in Malaysia. The results revealed that three months after the intervention mean differences between the groups were not significant for the Loneliness Scale, Anxiety Scale, and Depression Scale. In a Malaysian study, the control group participated in a group-based activity such as painting, drawing, and playing games over six week. ^38^ In the present study, the control group did not participate in any intervention programs. This can be one of the reasons for the differences between the findings of two studies. In addition, the participants in the Malaysian study were older than the participants in the present study, and their follow up was performed for three months after the intervention. Another study was conducted to determine the effect of spiritual group therapy on depression, anxiety, and stress in women with breast cancer. Results showed no significant effect of spirituality on stress and anxiety. They stated that stress occurred in cases of external conditions such as illness and as long as these stressors persisted, the person was still involved with stress. ^39^ However, the insignificant effect of spiritual counseling on stress and anxiety in their results may be due to different contents of counseling program and participating patients in the studies. They recruited cancer patients who may suffer from more mental problems due to the incurability. Moreover, in the present study, the counseling program was performed based on a specific instruction, but they focused mostly on spirituality and its behavioral aspects, and no instruction was followed. Generally, the differences in cultural, social, belief background, and different experiences toward spiritual and religious issues can lead to different results in the studies. 

This study has several limitations. We only studied the patients who were registered in MS society of Kashan city and the present sample size was small with a follow up of four weeks. Although the researcher emphasized that the patients should not disclose their counseling to others; however, this issue cannot be fully guaranteed. As the strength of this study, it is one of the few studies that assessed the effect of spiritual counseling on hope among patients with MS. 

## CONCLUSION

The present study concluded that spiritual counseling increased hope in patients with MS. Spirituality is needed for providing a comprehensive care, promoting the process of recovery, and improving the well-being of the patients. It is recommended that spiritual counseling should be used as a complementary method along with other counseling treatments to increase hope in patients with MS. There is a need for repetition of the current study with a larger sample size with more follow-up duration. Studies are needed with the sample drawn from various cultural, ethnical and socioeconomic subpopulations to strengthen the implications of the present findings. Further studies are recommended to be conducted to explore the effectiveness of spiritual counseling on hope in MS patients with severe disability. 
